# Increased Reporting of Infants Born With Congenital Syphilis to a Local Department of Public Health: A Quality Improvement Project

**DOI:** 10.1093/ofid/ofaf609

**Published:** 2025-09-29

**Authors:** John M Flores, Allyson Dewey, Brett K Palama, Yeo Won Ahn, Allison H Bartlett

**Affiliations:** Department of Medicine, University of Chicago Medicine, Chicago, Illinois, USA; Department of Pediatrics, University of Chicago Medicine, Comer Children’s Hospital, Illinois, Chicago, USA; Department of Infectious Diseases, Cook County Health, Chicago, Illinois, USA; Pritzker School of Medicine at the University of Chicago, Illinois, Chicago, USA; Department of Pediatrics, University of Chicago Medicine, Comer Children’s Hospital, Illinois, Chicago, USA; Department of Pediatrics, University of Chicago Medicine, Comer Children’s Hospital, Illinois, Chicago, USA; Department of Pediatrics, University of Chicago Medicine, Comer Children’s Hospital, Illinois, Chicago, USA

**Keywords:** congenital syphilis, infant, public health, public health surveillance, quality improvement

## Abstract

**Background:**

Congenital syphilis (CS) remains a significant public health concern. Current reporting guidelines may underreport cases where infants receive penicillin despite nonreactive rapid plasma reagin (RPR–) due to inadequate maternal treatment and delayed infant antibody seroconversion.

**Methods:**

This quality improvement project analyzed CS cases at a tertiary children's hospital from 2011 to 2022. A prospective intervention was implemented from September 2023 to February 2025 to improve the reporting of cases, including multidisciplinary communication, standardized electronic medical record data entry, and regular reminders.

**Results:**

Prior to the intervention, 154 infants were identified who had a case definition of CS per the Centers for Disease Control and Prevention and received penicillin administration therapy, with 107 of 154 (69.4%) RPR– and not reported to the local department of public health. At the end of the first intervention cycle, 44 infants were reported to the department of public health, with 15 cases (34.1%) being RPR– and successfully reported.

**Conclusions:**

This project highlights the limitations of RPR-based reporting for CS surveillance. The implemented intervention improved the reporting of cases, including those without maternal RPR positivity, thereby enhancing public health surveillance efforts.

## Teaser

In this quality improvement project, we successfully improved the reporting of infants diagnosed with and treated for congenital syphilis with nonreactive rapid plasma reagin blood test results to our local department of public health.

## Graphical Abstract

**Graphical Abstract:**
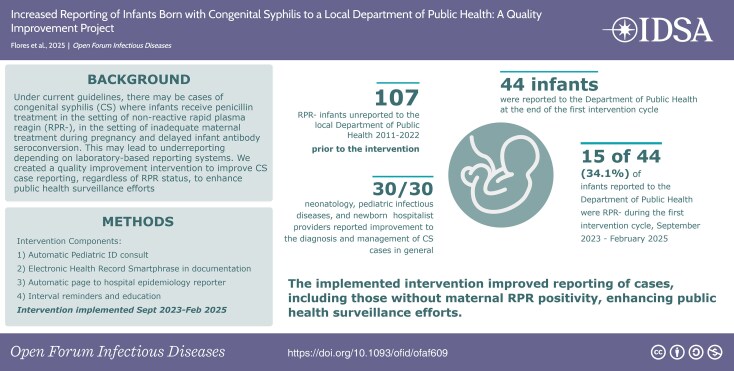
https://tidbitapp.io/tidbits/increased-reporting-of-infants-born-with-congenital-syphilis-to-a-local-department-of-public-health-a-quality-improvement-project?utm_campaign=tidbitlinkshare&utm_source=IO

In the United States, the number of congenital syphilis (CS) cases has increased dramatically, with a 755% rise documented from 2012 to 2021 [1–3]. In Chicago specifically, a 400% rise in cases from just 2019 to 2022 has been reported [4]. CS results from vertical transplacental transmission of *Treponema pallidum* from mother to fetus [5]. Morbidity and mortality outcomes associated with CS include stillbirth, neonatal death, deafness, neurologic impairment, and skeletal deformities [6–8]. Preventable factors, including a lack of appropriate testing and adequate treatment during pregnancy, have been identified as major contributors to the increase in cases [1]. Despite this dramatic increase, the underreporting of CS cases may be affecting our understanding of this ongoing epidemic. There is concern for underreporting of CS cases and stillbirths associated with syphilis due to nonadherence to Centers for Disease Control and Prevention (CDC) guidelines and classification schemes [9, 10].

Per the CDC 2021 sexually transmitted infection (STI) diagnosis and treatment guidelines, potential CS cases are classified by a variety of factors, including birthing mother diagnosis and treatment; physical examination findings, laboratory data, and radiographic evidence of syphilis in neonates; and ratio of maternal to neonate nontreponemal serologic titers [11]. Treatment of infants with a 10-day course of penicillin is indicated in scenarios where CS is “highly probable/confirmed” and when CS is deemed “possible” due to certain clinical contexts. Possible CS cases include neonates who are rapid plasma reagin nonreactive (RPR–) at delivery but whose parents had either inadequate treatment or no documented treatment. Despite these guidelines, heterogeneity exists in institutional reporting practices of CS, which has led to RPR– cases of treated neonates being underreported to departments of public health [9]. Treatment guidelines allow for treatment of an infant with possible CS with 1 dose of benzathine penicillin rather than a 10-day course, provided that close follow-up for symptom development and serologic monitoring can be ensured. Because of low follow-up rates in our institution [9], our practice is to treat these infants with 10 days of penicillin. Accurate reporting of CS cases is essential to characterizing the epidemiology of this current trend and effectively implementing interventions at the local and national levels.

## LOCAL PROBLEM AND AIM

Currently the Chicago Department of Public Health (CDPH) has standardized recommendations in place for reporting cases of CS, which are available to the hospitals reporting the cases and are mandated to be reported by Illinois state law. They offer guidance on definitions based on infant laboratory criteria, with or without symptomology, or maternal criteria based on adequacy of diagnosis and treatment during the pregnancy [12]. At our large tertiary care birthing center and children's hospital in Chicago, Illinois, infants with a seropositive RPR (RPR+) result are automatically notified from the microbiology laboratory to the infection control and prevention group, which in turn reports the cases to the CDPH to fulfill reporting requirements. In this process, any laboratory test with a RPR+ result or treponemal-specific antibody acts as a marker of a person with syphilis, regardless of age. The unique scenario where an infant may be RPR– due to delayed seroconversion but warrants a diagnosis and treatment of CS due to insufficient maternal treatment during pregnancy [11] was not considered upon creation of the protocol, which was implemented with the transition to an automated laboratory-based reporting system. A preliminary retrospective study utilizing *ICD-9* and *ICD-10* billing codes demonstrated that 19% of CS cases treated for 10 days with intravenous penicillin at our institution were RPR– at delivery. Therefore, these infants did not have the positive serologic test result to be automatically sent to the infection control and prevention group to screen the cases and, in turn, were not reported to the CDPH [9]. Therefore, basing reporting on RPR+ status alone may lead to significant underreporting of cases. Our aim was to create a quality improvement intervention to ensure that our institution has 100% accurate reporting of CS cases to the CDPH, regardless of RPR status, based on CDC diagnosis and treatment guidelines and Illinois state law [11].

## METHODS

### Context

The primary setting for this intervention was at Comer Children's Hospital at the University of Chicago Medicine in Chicago, a large quaternary referral center and high-volume delivery center with a large number of infants born with CS [9]. There have been no previous quality improvement initiatives related to CS reporting.

### Understanding the Context

Preliminary data were collected through electronic medical record (EMR) search functionality for all patients born between 2011 and 2022. Data were filtered by *ICD-9* code (090) and *ICD-10* code (A50.9) [13] and supplemented by penicillin administration for CS to capture those who may have had incomplete billing, all of which were then confirmed through chart review. Patients were excluded if they were not <30 days old, if they were billed for CS but did not have the disease, if there was no RPR status within the EMR, or if they were diagnosed and/or treated at an outside institution. Patients were then stratified as having CS with reactive or nonreactive RPR (RPR+ or RPR–). Each patient was also classified by a CS case definition per the CDC STI guideline [11] of less likely CS, possible CS, and proven/symptomatic CS based on review of the EMR. Infants with less likely CS are those born without symptoms to pregnant mothers who were diagnosed with syphilis during pregnancy, initiated adequate treatment with penicillin at least 30 days prior to delivery, and had no concern for reinfection. Infants with possible CS appear asymptomatic, but the birthing mother was untreated or inadequately treated, initiated treatment within 30 days of delivery, or had an unknown treatment status. Proven or symptomatic CS is when the infant has clinical, laboratory, or radiographic evidence of CS, regardless of maternal treatment history. The data were then cross-referenced with data reported and received at the CDPH.

### Study Design

Development of the intervention involved identifying key stakeholders in the project: pediatric infectious diseases (PedID) specialists, pediatric and newborn hospitalists, neonatologists, infection prevention and control providers, and representatives from the CDPH. Multidisciplinary meetings occurred on a biweekly basis until a consensus was formed regarding the various components.

**Figure 1.**
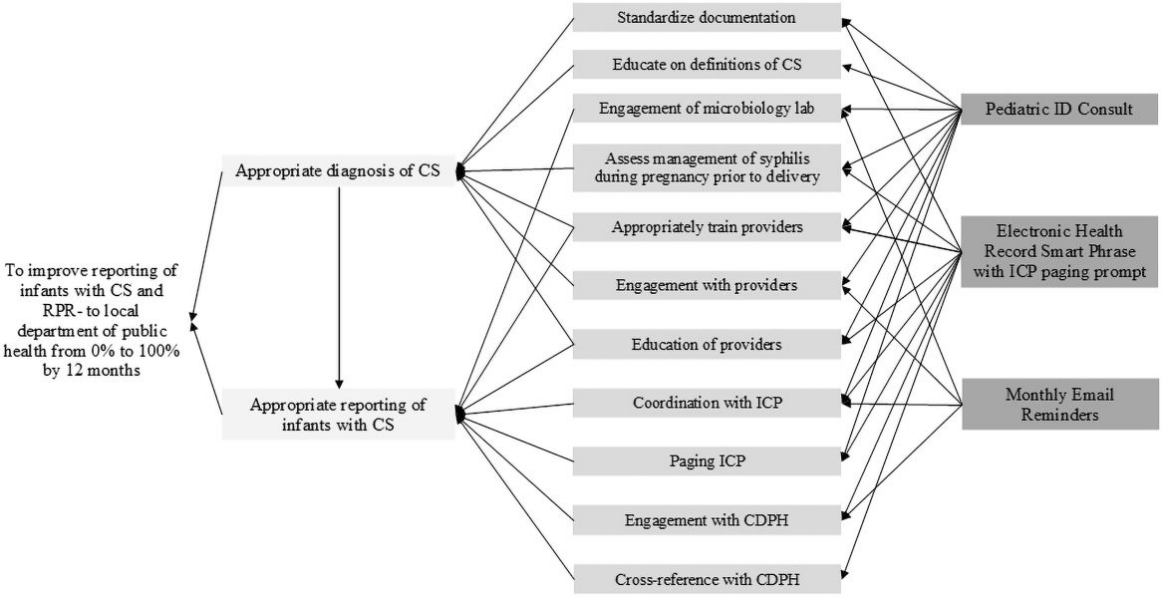
Action effect diagram for initiative to improve reporting of infants with congenital syphilis to the Chicago Department of Public Health. From left to right, the text columns correspond to the shared aim, major contributing factors, secondary contributing factors, and implementation activity factors. CDPH, Chicago Department of Public Health; CS, congenital syphilis; ICP, infection control and prevention providers; ID, infectious diseases; lab, laboratory.

This study is based on an action effect method, which is a facilitated approach to developing an action effect diagram—in this case, a visual representation of the program theory for the quality improvement initiative. This diagram was utilized to allow for articulation of the following: (1) the overall aim, (2) the potential interventions tested in an attempt to achieve this aim, (3) the hypothesized cause-effect relationships linking interventions to the aim, and (4) the measured concepts that link to the cause-effect chains to support evaluation [14] (Figure 1). The study was undertaken from 1 September 2023 through 28 February 2025.

### Intervention

The intervention included 4 primary components. The first was the formalized expectation to page the PedID on-call team for all infants with concern for maternal syphilis exposure to assist in the diagnosis and management of the case. The second was to create an EMR smart phrase in the PedID consult documentation to standardize data presentation for clinical and reporting purposes (Table 1) and within the text to prompt the PedID providers to directly contact infection control providers involved in reporting the cases of CS to the CDPH through the Chicago Health Information Management System [12]. The third was to standardize *ICD-10* diagnosis coding practices, as review of our baseline data of penicillin administration demonstrated variability in *ICD-9* and *ICD-10* coding practices. Fourth, email reminders were sent monthly to the key stakeholders of the components of the PedID consult, EMR smart phrase, and billing practices. Infants discharged from their initial birthing inpatient hospital stays with maternal syphilis exposure and a diagnosis that included a CS case definition per the CDC STI guideline [11] were then prospectively followed to determine if their cases were reported to the CDPH. At the 6-month intervention period, a quantitative postintervention survey was sent to identified stakeholders to determine balancing measures to assess for unintended consequences, the acceptability of the intervention, and its impact on clinical care related to CS diagnosis and management.

**Table 1.** Electronic Medical Record Smart Phrase

Congenital Syphilis Checklist	Provider Action
**Maternal**	
RPRs with dates	[Fill in]
Treatment (penicillin specific, ×3 dose IM PCN, etc)	[Fill in]
Adequate treatment?	Yes/no [Bold or delete]
**Infant**	
RPR	[Fill in]
CBC with differential	[Fill in]
CMP or LFTs	[Fill in]
Diagnosis	Symptomatic/proven, possible, less likely, unlikely [Bold or delete]
Treatment	×1 dose IM benzathine PCN, 50 000 U/kg
	10 d IV aqueous PCN, 50 000 U/kg, every 12 h days 1–7, every 8 h days 8–10
	[Delete option not used]
Infection control paged?	[Fill in]

Abbreviations: CBC, complete blood count; CMP, complete metabolic profile; IV, intravenous; IM, intramuscular; LFT, liver function test; PCN, penicillin; RPR, rapid plasma reagin.

### Measures

The primary outcome measure is the number of infants who were diagnosed and treated with a CS case definition per the CDC STI guideline [11], had a seronegative RPR blood test result, and were reported to the CDPH. A positive change was a confirmed receipt of the case from our infection control and prevention team to the CDPH through cross-matching with the CDPH syndemic bureau database. The primary author had professional roles within the birthing hospital and CDPH, which allowed him access to these data. Through the postintervention survey, secondary process measures were collected, which act as fidelity measures for how well our planned intervention was accepted and applied as intended. Measures included confirmed clinical care of infants born with maternal syphilis exposure; utilization of the PedID consult service; perceived impact of PedID consult service on clinical diagnosis, management, and follow-up of infants with CS; confirmed knowledge of the EMR smart phrase; and clinical impact of the EMR smart phrase.

### Analysis

Data for the primary outcome were plotted on a statistical process control (SPC) X-chart. They were analyzed by the quality improvement macros add-on application to Microsoft Excel with traditional run chart and SPC chart rules. Specifically, shifts in the data over time—defined as either ≥6 data points reflecting a clear increasing trend or at least 1 data point above the upper control line—were assessed and monitored for since implementing the intervention. Basic frequency statistics were used to analyze process measures.

## RESULTS

### Primary Outcome Measure

Between 2011 and 2022, 154 infants were diagnosed with and treated for symptomatic/proven, possible, or less likely CS. Most infants treated (107/154, 69.4%) were RPR–, and 41 of 107 (38.3%%) were RPR– and received >1 day of penicillin (Table 2). It was determined through cross-referencing with the CDPH database that none of the 107 (0%) RPR– cases had been reported to CDPH.

**Table 2.** All Infants With CS Stratified by RPR Results: 2011–2022 and September 2023–February 2025

RPR Status	1 d of PCN	>1 d of PCN	Total
All infants with CS, 2011–2022			
RPR−^a^	66	41	107
RPR+	2	45	47
RPR− and RPR+	68	86	154
All infants with CS, Sep 2023–Feb 2025			
RPR− ^b^	6	9	15
RPR+	4	25	29
RPR− and RPR+	10	34	44

Note that all infants with CS from September 2023 to February 2025 followed initiation of the intervention.

Abbreviations: CS, congenital syphilis; PCN, penicillin; RPR, rapid plasma reagin.

^a^RPR– cases were not reported to the local or state department of public health for processing.

^b^RPR– cases were all reported to the local or state department of public health for processing.

**Figure 2.**
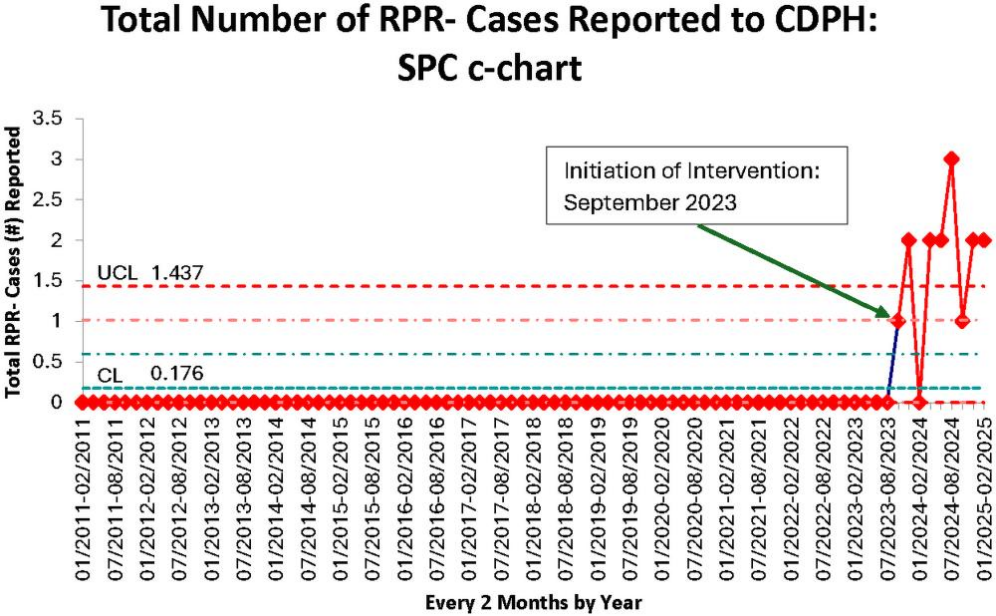
Statistical process control (SPC) c-chart. This figure represents the changes in reporting of infants with nonreactive rapid plasma reagin (RPR–)from the beginning of the preliminary data collection (January 2011) through the initiation of the intervention (September 2023) and the end of the first cycle of the intervention (February 2025). The preintervention period represents the time prior to the intervention, where zero RPR– cases were reported to the Chicago Department of Public Health. The bundled columns represent 2-month groupings. The x-axis is composed of clumped time units (month/year through month/year). The y-axis is number of RPR– cases reported to the Chicago Department of Public Health. The upper control limit (UCL) is the threshold above which the reported RPR– cases are considered to show special cause variation. The center line (CL) is the mean value of the RPR– cases being measured. The lighter red and lighter blue lines signify two sigma limits.

During the intervention period, 44 infants were prospectively diagnosed with a CS case definition per the CDC STI guideline [11] and received penicillin therapy, regardless of RPR status (RPR– and RPR+). Of the 44, 15 were RPR– (34.1%) and received some form of penicillin therapy, whereas 9 (20.5%) were RPR– and received 10 days of penicillin for a diagnosis of symptomatic/probable or possible CS (Table 2). It was determined through cross-referencing with the CDPH database that all the infants who were RPR– (15/15, 100%), regardless of duration of penicillin use, had forms submitted from our infection control and prevention team for reporting purposes. No RPR– cases were missed despite the intervention. Additionally, all infants diagnosed with CS, regardless of RPR status (44/44, 100%), were reported successfully. An SPC c-chart was created that noted a center line value of 0.176, an upper control limit of 1.437, and 6 total measurements above the upper control limit (Figure 2). Additionally, upon retrospective review of *ICD-10* billing code auditing, it was determined that all infants who were diagnosed with CS and received treatment with penicillin (44/44, 100%) received an appropriate *ICD-10* billing code.

### Process Measures

Thirty neonatology, PedID, and newborn providers completed the midintervention survey on the quality improvement process at the 6-month mark. All 30 (100%) responded that the intervention improved the diagnosis and management of CS cases in general. In total, 28 (93.3%) indicated that the PedID service improves the follow-up of an infant with concerns for CS. Furthermore, 26 (86.7%) providers responded that a PedID consult should be requested for maternal syphilis exposure to evaluate for CS “every time” (n = 17) or “most of the time” (n = 9). The survey data were disseminated to PedID, neonatal, and newborn nursery medical providers. In the final question of the survey, which had an open qualitative section, there were no adverse or unintended consequences reported by survey respondents. The processes of this intervention directly affected a hospital-wide CS pathway that was created in September 2024, with the inclusion of an automatic page to the on-call PedID provider for all infants exposed to maternal syphilis to discuss diagnosis and management of the patients. To date, there has been a continued success, with 100% reporting of RPR– cases.

## DISCUSSION

In this quality improvement project, we successfully improved the reporting of infants diagnosed with and treated for CS with RPR– blood test results to our local department of public health. Furthermore, we were able to create a feasible intervention that has been accepted by multiple pediatric specialties that care for infants with CS within the Comer Children's Hospital setting. We determined through our provider assessment survey that the inclusion of a PedID consult service was perceived by newborn nursery and neonatology providers to enhance the diagnosis, management, and follow-up of infants with maternal syphilis exposure. Additionally, based on our SPC chart results, there appears to be a significant improvement in CS reporting of infants with RPR– serologies and a CDC case definition of CS, likely not due to random variation or chance [11].

As the incidence of CS continues to rise [1], a comprehensive understanding of its epidemiology is crucial to inform effective public health interventions. By identifying the underlying factors contributing to the resurgence of this preventable disease, we can develop targeted strategies to reduce transmission and eliminate CS. The diagnosis of CS may be clinically challenging. The 2021 CDC STI guidelines note that CS can be classified into 4 distinct categories scenarios that determine the treatment course: symptomatic or proven, possible, less likely, and unlikely [11]. However, diagnosis of CS can be difficult because maternal nontreponemal (RPR) and treponemal immunoglobulin G antibodies can be transferred through the placenta to the fetus in an unpredictable manner, complicating the interpretation of reactive serologic tests for syphilis among infants who are <30 days of age, which may not necessarily warrant treatment or diagnosis [15, 16]. Additionally, using RPR abnormality as the basis for electronic reporting of CS to departments of public health and then to the CDC undercounts true incidence. There are scenarios where the infant may be asymptomatic but still warrant a diagnosis of CS with penicillin treatment, dependent on the inadequacy of maternal treatment and/or maternal and nontreponemal serology results, even in the setting of a negative infantile RPR. In certain settings, these infants are diagnosed and treated for CS but may not be reported to local or national public health authorities [9, 11]. Scenarios such as this have been described, with recent CDC and Illinois Department of Public Health CS response initiatives, based on missed opportunities identified by the CDC in CS prevention [1, 17, 18]. While such interventions could cause overreporting at the local level, there are processes in place through local departments of public health and the CDC that limit this risk by finalizing case determinations regardless of reporting volume. This balancing measure of number of cases reported to the local department of public health that the CDC adjudicated to be not CS were unable to measured in this study, but we are planning to pursue these data in ongoing iterations of this study.

To add further complexity to the scenario, the most utilized billing system, the *ICD*, does not have these CDC diagnostic definitions as codes but rather more broad terms such as “congenital syphilis, unspecified” (*ICD-10-CM* A50.9) as well as symptom-specific codes such as “early congenital syphilitic pneumonia” (*ICD-10*-*CM* A50.04s) [13]. These various barriers may lead to significant heterogeneity in diagnosing, managing, and reporting this increasingly prevalent disease, which reinforces the importance of the findings in our study and the potential for utilizing standardized processes such as ours, which include a multidisciplinary approach and the incorporation of EMR smart phrase technology.

Future directions of this project include continued follow-up and monitoring in subsequent cycles to determine if the enhanced reporting to the CDPH has been sustained, by observing longitudinal data beyond the 17-month intervention reported in this article. A revised intervention assessment survey will be distributed to CDPH epidemiologists on the receiving end of the CS case reports to assess acceptability of the intervention. A follow-up rigorous qualitative study involving individual interviews and focus groups of the pertinent stakeholders involved in the intervention may be performed to detect nuances regarding the intervention not detected in the quantitative postsurvey analysis. This intervention may be replicated in other birthing and/or children's hospitals to determine generalizability to other settings outside of our single center. Additional studies will analyze whether the enhanced reporting led to other downstream effects, such as better follow-up, more timely treatments, and improved health outcomes on a patient level and population level.

Our study has multiple limitations. Utilizing *ICD* codes may lead to underinclusion due to coding errors or to infants being treated or diagnosed elsewhere. We attempted to overcome this through excluding all infants treated elsewhere and by utilizing penicillin administration data, as it is exceedingly rare to use penicillin in an infant outside of CS treatment [10, 11]. While using pharmaceutical data may lead to overinclusion, almost all infants noted had expert pediatric hospitalist or PedID involvement in diagnosis and management to counteract this potential bias. We recognize that because of the nuances and ambiguity of certain CDC case definitions [11], it is up to the interpretation of the provider whether an infant warrants diagnosis and treatment of CS, as opposed to simply maternal syphilis exposure, which may lead to differences in reporting the infants who are RPR–. To overcome this, multiple members of the study team, who are PedID providers, audited each chart and confirmed diagnostic accuracy of those infants who should or should not have been reported, per the diagnostic criteria created by the CDC [11].

Additionally, while we were able to improve our institutional reporting and confirmation of the receipt of our cases from the CDPH, we were unable to obtain the final case determination outcomes by the CDPH, which may have been different from our own clinical evaluation. We attempted to overcome this through the integrated PedID consult service to provide the most consistent and evidence-based recommendations. An additional limitation is the potential for resistance to adopt the intervention among primary and consult providers. We were able to overcome this through preliminary multidisciplinary meetings in the development of the intervention, and we sought approval in the initial introductions to the interventions from stakeholders. This is a necessary component to allow for sustainability and ongoing adjustments as required. Within our intervention survey distributed to the clinical parties involved, we did not find any adverse or unintended consequences of the project, but we were unable to gather this information from the epidemiologists on the receiving ends of our reports from the CDPH. Additionally, we were unable to compare the downstream effects from our project, such as improved health outcomes for these infants, particularly as compared with RPR– cases not reported in our preliminary data extraction.

Another limitation is a lack of generalizability, as our intervention may be difficult to operationalize at hospitals without PedID services or EMR customization capacities. There is a significant shortage of PedID providers [19], and this may affect diagnosis, management, and reporting of CS. This could be overcome through public health webinars on proper reporting practices or utilization of centralized reference guidelines [11] or by expanding on-call telephone services specific to assisting in the clinical management of CS [17, 19]. Similarly, other birthing hospitals may have different forms of CS reporting that do not involve RPR-triggered protocols, for which this intervention would then not be applicable. Finally, follow-up of this quality improvement project could be the development of a generalized protocol, regardless of PedID consult and/or EMR accessibilities, that could be replicated in birthing hospitals to ensure proper reporting.

## CONCLUSION

In this quality improvement project, we successfully improved the reporting of infants diagnosed with and treated for CS to our local department of public health. The intervention was accepted by stakeholders, including newborn and pediatric hospitalists, neonatal providers, and PedID specialists. As we confront the rising incidence of CS, it is critical that we have a complete understanding of the epidemiology, which may lead to increased support for public health initiatives focused on decreasing and eliminating CS.
